# Contextual challenges and impacts on the surgical ecosystem in Chiapas, Mexico: A qualitative study

**DOI:** 10.1371/journal.pone.0321969

**Published:** 2025-04-29

**Authors:** Zachary Fowler, Amina Rahimi, Alejandra Cantu Aldana, Tarsicio Uribe-Leitz, Fernando Carrillo-Villaseñor, Lina Roa, Sarah K. Hill, Valeria Macias, Manuel Castillo-Angeles, Amanda J. Reich

**Affiliations:** 1 Department of Surgery, Donald and Barbara Zucker School of Medicine at Hofstra/Northwell, New Hyde Park, New York, United States; 2 Center for Surgery and Public Health, Department of Surgery, Brigham and Women’s Hospital, Harvard Medical School and Harvard T. H. Chan School of Public Health, Boston, Massachusetts, United States; 3 Harvard Medical School, Boston, Massachusetts, United States; 4 School of Social Work, Boston College, Chestnut Hill, Massachusetts, United States; 5 Program in Global Surgery and Social Change, Harvard Medical School, Boston, Massachusetts, United States; 6 Department of Plastic and Oral Surgery, Boston Children’s Hospital, Boston, Massachusetts, United States; 7 Chair of Epidemiology, School of Medicine and Health, Technical University Munich, Munich, Germany; 8 Instituto Mexicano del Seguro Social “El Marques”, Querétaro, Mexico; 9 Department of Obstetrics and Gynecology, University of British Columbia, Vancouver, Canada; 10 Department of Surgery, The University of Toledo, Toledo, Ohio, United States; 11 Compañeros en Salud, Chiapas, Mexico; 12 Division of Trauma, Burn, and Surgical Critical Care, Department of Surgery, Brigham and Women’s Hospital, Harvard Medical School, Boston, Massachusetts, United States; Institute of Public Health from Guanajuato State, MEXICO

## Abstract

Chiapas is a state in southern Mexico that faces significant challenges in healthcare delivery. Strengthening the surgical system requires a comprehensive understanding of all health system domains and the contextual factors that influence care delivery. This study used qualitative methods to identify factors related to both gaps and successes in surgical care in Chiapas, Mexico. Semi-structured interviews were conducted with 23 participants at 15 public and private hospitals. Participants consisted of nurses, physicians, surgeons, and hospital administrators. Interviews were transcribed, and a codebook was developed and applied to all interviews. Recurring themes were identified and described using thematic analysis. Four themes characterizing the challenging context through which care is delivered were identified: referral system challenges, workforce shortages, insufficiencies in perioperative and nonoperative care, and waste and mismanagement of resources. Three themes related to innovations and workarounds were identified: efforts to maximize resources and reduce waste, strategies to reduce language barriers, and planning to account for clinical needs in situations of limited access and emergencies. Gaps and challenges within the surgical system of Chiapas lead to challenges in care delivery across all domains of the health system. However, several solutions have emerged among local providers. Insight into these factors can be used in planning efforts to improve access to safe and effective surgical care.

## Introduction

Global surgery has seen a recent period of rapid growth and change following several key events. In 2015, the Lancet Commission on Global Surgery published its report, *Global Surgery 2030*, which established a clear vision for global surgery and aimed to provide structure to a heterogeneous field [1]. The World Health Assembly Resolution 68.15 was also passed, which provided a mandate for the World Health Organization (WHO) and Member States to strengthen surgical and anesthesia care as a component of universal health coverage (UHC) [2]. These milestones reflect a paradigm shift in global surgery from vertical programs to comprehensive health system strengthening.

Chiapas is a state in southern Mexico with the highest level of poverty in the country in 2020 [3]. Despite the creation of a UHC program, large health disparities have been noted in this region, and there have been efforts to mitigate them both from the Mexican government and from nongovernmental organizations [4–10]. In Mexico, healthcare is delivered through three distinct systems: social security institutions for beneficiaries with formal employment, safety net institutions for those without formal employment, and private hospitals. The largest social security institutions are the Mexican Social Security Institute (IMSS) and the Institute for Social Security and Services for State Workers (ISSSTE) [11]. Healthcare services for those without formal employment were previously administered through the Seguro Popular program but have now transitioned to IMSS-Bienestar, which has been expanded [12]. Recently, the National Development Plan 2025–2030 was launched and includes specific plans to improve healthcare. Among the objectives outlined are plans to consolidate IMSS-Bienestar, modernize hospitals and health centers, and develop new facilities within the IMSS, IMSS-Bienestar, and ISSSTE systems [13]. In Chiapas, 80% of residents were enrolled in the safety net system, which allows them to access free or low-cost service [4–6]. This fragmented system also brings unique challenges that require targeted solutions.

Improving surgical care in Chiapas and in other similar settings will require a comprehensive approach that includes all components of the surgical ecosystem. Several frameworks have been proposed to strategically improve surgical care. For example, the concept of the National Surgical, Obstetric, and Anesthesia Plan (NSOAP) is based on six health system domains and was adapted from the WHO Health System Building Blocks framework [14]. Another framework proposed by the WHO Regional Office for the Western Pacific utilizes system inputs and processes in the setting of operational shifts [15]. To implement changes in rural Mexico using these frameworks, a deep understanding of the system is needed. And while available quantitative data provide information about infrastructure, services, and outcomes, qualitative research will provide insight into the root causes of these system challenges. Thus, the aim of this study was to identify themes regarding gaps and solutions to surgical care provision in Chiapas, Mexico.

## Methods

### Methodology

This study represents the qualitative component of a comprehensive assessment of surgical capacity in Chiapas. The quantitative results have been reported elsewhere [16,17]. Institutional Review Board approval was granted by the Chiapas Ministry of Health Committee on Bioethics (approval number 5003/8777). This study adhered to the Consolidated Criteria for Reporting Qualitative Research (COREQ).

### Setting

The state of Chiapas is divided into ten health jurisdictions by the Ministry of Health [18]. Hospitals in the public sector are classified according to three designated levels of care: *básico comunitario* (primary*), general* (secondary)*,* and *regional* (tertiary) [18]. Private hospitals do not receive level designations and vary widely in size and scope of services [16]. All facilities in Chiapas providing cesarean delivery, laparotomy, or open fracture repair were identified using public databases [19]. The research team visited one public hospital corresponding to each level within each jurisdiction for data collection (FCV, EM, ZF). All private hospitals were invited to participate.

### Instruments

The interview guide was developed by the Program in Global Surgery and Social Change in partnership with the World Health Organization as a tool for research to aid in development of National Surgical, Obstetric, and Anesthesia Plans as outlined by the Lancet Commission on Global Surgery. It accompanies a quantitative assessment tool and was developed through Delphi consensus by a multidisciplinary team [14]. The interview guide has been used for qualitative research in at least 9 countries in Asia, Africa, and the Americas [20–24]. The interview guide was initially used in two interviews that were reviewed by the research team to ensure applicability in the local context and included unique guides tailored for nursing staff, anesthesia providers, surgeons and obstetricians, and hospital directors. All interview guides elicited information about infrastructure, workforce, service delivery, financing, and information management [20,21,25].

### Subjects

During facility visits, the study aim and methodology were described to hospital leadership, who then asked for volunteers to participate in interviews. We sought interviews with surgeons, anesthesiologists, obstetricians, nurses, and hospital directors. Verbal informed consent was obtained in audio recordings for all interviews which was witnessed by a second member of the research team. Participant recruitment began on October 9, 2019 and was completed on December 18, 2019.

### Data collection

Interviews were conducted in Spanish by one Mexican medical student (FCV) and one US resident physician (ZF). One of the interviewers (ZF) had experience in qualitative research, and both received additional training for this study by an academic investigator specializing in qualitative methods (MC-A). The interviewers had worked in hospitals in Chiapas prior to the initiation of the study and were familiar with the local health system but did not know the participants. Interviews were conducted in-person at the hospitals in a private room which was chosen by the participant. Interviews ranged from 20 to 78 minutes in duration (mean = 42; SD = 16). Audio recordings were digitally transcribed for analysis.

### Data analysis

Using a grounded theory approach, the data were analyzed comprehensively and inductively. Thematic analysis was used until saturation was reached. The coding tree was developed using three transcripts that were translated to English. It was developed by academic doctoral-level researchers specializing in qualitative methods (AJR, MC-A). Coding was done in weekly meetings by them as well as members of the research team who were familiar with the local health system (TU-L, AC). The codebook was revised iteratively and ultimately consisted of 15 codes as well as subcodes. The final codebook was applied to all transcripts. Two coded transcripts were coded by a third researcher (MC-A) to confirm reliability. The research team also reviewed all coded transcripts together and resolved disagreements in the application and interpretation of codes through weekly research team meetings.

## Results

We conducted 23 interviews at 15 hospitals in Chiapas across eight jurisdictions. Participant characteristics are listed in Table 1. A total of seven themes were identified, which were categorized in two groups: contextual challenges and innovations (Table 2).

**Table 1:** Participant characteristics.

Participant	Title	Sex	Hospital type	Setting
1	Nurse	Female	Public (1st level)	Rural
2	Anesthesiologist	Female	Public (1st level)	Rural
3	Director	Female	Public (1st level)	Rural
4	Anesthesiologist	Male	Public (2nd level)	Urban
5	Anesthesiologist	Male	Private (for profit)	Urban
6	Anesthesiologist	Male	Private (for profit)	Urban
7	Nurse	Female	Private (for profit)	Urban
8	Nurse	Female	Private (for profit)	Urban
9	General surgeon	Male	Private (for profit)	Urban
10	General surgeon	Male	Private (for profit)	Urban
11	Director	Male	Private (for profit)	Urban
12	Director	Male	Private (for profit)	Urban
13	Director	Female	Private (nonprofit)	Rural
14	Nurse	Female	Private (nonprofit)	Rural
15	Nurse	Male	Private (nonprofit)	Rural
16	OBGYN	Male	Private (for profit)	Urban
17	Orthopedic surgeon	Male	Private (for profit)	Urban
18	General surgeon	Male	Public (1st level)	Rural
19	Nurse	Female	Public (1st level)	Rural
20	Director	Male	Private (for profit)	Rural
21	General surgeon	Male	Public (2nd level)	Rural
22	General surgeon	Male	Public (2nd level)	Urban
23	Director	Male	Private (for profit)	Urban

**Table 2:** Themes identified.

Contextual challenges	Innovations and workarounds
• Referral system challenges • Workforce challenges and dependence on volunteers • Deficiencies in perioperative and nonoperative aspects of surgical care • Inefficiencies and ineffective management	• Efforts to maximize resources and reduce waste • Strategies to reduce language barriers • Planning to account for clinical needs from access to emergency situations

### Challenging context

The context through which care is delivered has substantial influence on the ability to provide effective care. These contextual factors can be understood through the lens of the Social Ecological Model, which views health outcomes as the result of complex interactions between structural, interpersonal, and individual factors [26]. Participants described diverse factors that contributed to this challenging context, including issues associated with the referral system, workforce shortages, insufficiencies in perioperative and nonoperative care, and waste/mismanagement of resources.

### Referral system challenges

Surgical providers frequently reported difficulties referring patients from rural or district hospitals to larger hospitals. The challenges originate along a continuum of acute care, within the referring hospital, during transportation, and at the receiving hospital. These challenges were attributed to deficits in infrastructure, supplies and equipment, and human resources at first level hospitals referring patients. This required a large number of referrals even for basic surgical care that is intended for first level hospitals. Additionally, transportation challenges were frequently cited and include both access to transportation and safety during transport. One participant shared an experience with the ambulance system, “The ambulance didn’t have an oxygen tank, you have to provide your own, and the safety mechanism to secure the bed to the floor didn’t work, so the bed was moving during transport…In fact, they told us that the ambulance wouldn’t make it to Villaflores (*city where the receiving hospital is located*), so we transferred to another ambulance on the highway. In no way can that be safe.” Additionally, ambulances are sometimes unavailable and patients who require monitoring by a health professional are required to use taxis for transfer between hospitals. One participant described the case in which a patient with peritonitis had to be sent to another hospital in a taxi because the only ambulance was already in use, and by the time the taxi had arrived at the transfer hospital, the patient had died. This unfortunate incident illustrates the intersection between limited resources at referring hospitals and transportation limitations.

Furthermore, receiving hospitals frequently deny requests from small hospitals because they do not have beds available. Patients must then choose between waiting for a bed to become available or seeking care at a private hospital and paying out of pocket. If there are beds available, there may not be supplies, and the receiving hospitals then send patients to purchase supplies. Providers also described the added challenges that referrals present for patients. Patients from rural communities often cannot afford transportation costs. Several participants discussed the measures taken by the patient population to pay for medical care. As one stated, “They sell their animals, be it cattle or chickens, or whatever they harvest.” Another participant noted that even this is a considerable challenge for patients when the weather is problematic for farming, “We can’t even grow maize or coffee because it hasn’t been raining.” In addition to a financial challenge, providers also described the emotional challenge created when indigenous patients are transferred outside of their region, “Referrals are very, very exhausting physically, mentally, and financially, not only for the hospital but also for them because they are anxious about not speaking Spanish. Some already speak, they already understand, but they don’t have the ability to express themselves fully, and then removing them completely from their environment, they feel totally lost, with a lot of anguish.”

### Workforce shortages and dependence on volunteers

Workforce shortages were frequently reported as a challenge unique to Chiapas compared to more metropolitan states in Mexico. Providers attributed shortages in the region to doctors trained in Mexico not wanting to work in these communities, given the rural setting and resource constraints. One participant noted, “Specialists will not come here. Generally, specialists are young, and they get married and have kids and start thinking, where will I educate my child?” There are many things that you can’t find in this region. Also, we don’t have the economic resources to pay a specialist. The hospital functions mainly by donations and very minimal payments by patients when they can.” All of these factors present barriers to successfully recruiting specialists who stay in the region long-term. This further perpetuates a reliance on volunteers with sporadic and short visits to the region. One participant shared that the surgeon at the hospital at the time of the interview was a volunteer from another country on a 2-week-long campaign who operated on 110 patients during this time period. Another factor compounding workforce shortages is regional conflict and concerns for personal security. Providers described feeling unsafe and threatened in their daily work. One participant described a situation involving a visiting specialist who was kidnapped and assaulted while traveling to the hospital. Another stated, “yes they have come and threatened us, that if we don’t do what they want, they will kidnap us. So that’s one of the greatest limiting factors, that many people think of this as a conflict zone.” Thus, participants suggested that Chiapas as a region would need to undergo significant economic development to be able to pay the salaries of specialists, to provide specialists with the medical resources they need to practice, and so that specialists could raise their children in Chiapas.

These workforce shortages can make it challenging to adhere to national standards of care. One provider shared his frustration with this, “If a woman with hemorrhage from uterine atony comes, and I have to perform a cesarean or hysterectomy…we’re in the 21st century, I’m not a gynecologist, I’m a general surgeon and a cesarean should be done by a gynecologist, and there should be a pediatrician, and there should be blood [products], but there’s nothing!”

### Deficiencies in perioperative and nonoperative aspects of surgical care

A salient theme amongst providers interviewed was that even when a trained surgeon is present, preoperative and postoperative care is often insufficient to provide comprehensive care. This has included chemotherapy and radiation for patients requiring operations for cancer, as well as intensive care for critically ill patients after their operation. Surgeons are faced with the decision to provide surgery or forgo care because it would not be adequate to treat the patient’s condition. One surgeon described performing a thoracotomy and decortication for tuberculous pleurisy but felt that he was taking a risk because his facility could not provide adequate postoperative care for patients undergoing thoracic surgery. Another surgeon described a patient who required a colostomy, but postoperatively was not able to purchase the supplies needed to manage the colostomy at home. Describing one such situation, a surgeon said, “It has happened that we operate on a patient with closed thoracic and abdominal trauma, we place the chest tube, we perform laparoscopy, a splenectomy, etc., and then we have to send him to another hospital. Why would we do this when the nearest hospital is 4 hours away? We don’t have the resources for intensive care management of surgical patients.”

### Inefficiencies and ineffective management

Inefficiencies within the healthcare system were frequently described, and these spanned all levels of management, from health system administrators to surgeons and hospital directors. Investments in the health system were often piecemeal and did not result in improved capacity to provide surgical care. For example, new hospitals were built, and facilities were upgraded, but after the projects were completed, these hospitals faced large equipment shortages that limited the care they could provide. As one provider describes, “they’ve just remodeled the hospital, but there are many more practical deficiencies, there are many shifts without a surgeon available.” Other healthcare workers described working in facilities that hired new providers when there wasn’t adequate space or infrastructure for them, such as operating rooms or blood banks, and after their hire they were not able to work. This resulted in patients being sent to higher level hospitals.

Poor planning was also cited as a source of inefficiency. Several providers described experiences in which a hospital hired only part of a surgical team. For example, hospitals would have an anesthesiologist but no surgeon at certain times. Or they may have a surgeon but no nursing staff for the operating room. Without a complete team (*e.g.*, surgeon or obstetrician, anesthesiologist, and surgical nurse) they were not able to schedule surgeries and had to refer patients to other hospitals. This would leave highly trained specialists unable to use their skills. Additionally, this shift in patient volume from district hospitals to referral hospitals creates a bottleneck at the referral hospitals. One surgeon at a large public hospital described that due to the large number of surgical patients referred who need urgent care, elective procedures such as hysterectomies are significantly delayed.

Providers also discussed a substantial amount of waste within the system that occurs while many hospitals are under-resourced. One hospital director described how different surgeons in the same hospital had different equipment or suture preferences, and multiple types of the same product are purchased, with some going to waste. Private hospital providers reported that they discard any expired medications because they don’t have enough demand. Meanwhile, the public hospitals don’t have enough medications.

One provider described how workforce shortages and waste interact together to compound inefficiencies and waste. A high turnover of surgeons in rural settings leads to an accumulation of that surgeon’s preferred materials and equipment that ultimately needs to be disposed of when that surgeon leaves and a new surgeon makes different requests. A nurse in a private hospital describes this: “Here at the hospital, physicians and surgeons have come … they only stay for a certain amount of time. Then those surgeons ask for sutures. Let’s say a surgeon asks for one type of suture, for example, chromic or Vicryl. Well then that surgeon leaves and another comes that doesn’t want that suture, they ask you for another type of suture. So that’s how we end up accumulating these sutures.”

### Innovations and workarounds

Despite the challenges that exist in Chiapas, surgical providers have developed innovations and methods to work across some of these barriers. Innovations included agreements between surgeons and hospitals to practice in other public hospital systems if patients in need had presented elsewhere. Several hospital directors also described informal systems of sharing resources between hospitals. Surgeons organized campaigns to manage the backlog of elective cases and make room for emergent cases. Various providers also discussed the importance of understanding the cultural context to better serve the population in Chiapas.

### Efforts to maximize resources and reduce waste

Providers discussed shortages in materials driven by costs, regional supply shortages requiring hospitals to order materials from different states, reliance on donations, and high turnover of surgeons. Despite these challenges, providers are strategizing to maximize resources and reduce waste without compromising patient safety. Several hospital directors described informal systems of sharing resources between hospitals, often using mobile instant messaging platforms. As one surgeon in a public hospital describes, “There’s a referral form. The patient’s case is described, the reason they’re being sent. We accept it by WhatsApp or email. Obviously sometimes email is more difficult due to the internet problems, sometimes WhatsApp is much faster and is the way they can answer us to accept the patient at another hospital.”

Hospitals are also investigating which medications, materials, and equipment can be safely used beyond their stated expiration date. One provider described that while previously individuals were making decisions regarding extending expiration dates for materials on a case-by-case basis, those decisions now come directly from hospital leadership. Speaking on the impact of these workarounds, one participant shared, “the patient doesn’t even have enough to eat and even less to be transferred to another hospital or some medicine that we don’t have, some supplies. So what do we do? The only way is to work together, get it somewhere else, talk to another hospital, borrow it.”

### Strategies to reduce language barriers

Frequently, participants described the importance of multilingual staff in communicating with patients and improving patient satisfaction. There are five different languages spoken in the region, including Tzeltal, Tzotzil, Ch’ol, Zoque, and Tojolab’al. At some hospitals, providers speak Spanish and English, but not an indigenous language. Thus, there is an opportunity to improve care by increasing the number of bilingual staff and providing language learning resources. Some providers at private hospitals described policies to intentionally hire staff who speak an indigenous language, “even if at 50% proficiency”. One provider who is bilingual in Spanish and Tzeltal shared that he is immediately accepted by patients when they hear him talking to them in their language. Language concordant care allows for a more detailed and precise level of understanding of the care being provided or recommended. Providers underscored the long-term impacts that language concordant care and patient trust might have in the future for preventing patients presenting emergently or at advanced stages of disease.

Interestingly, one provider described a licensing program in indigenous languages that was opened. He was the only person who enrolled, and the program was closed the following year due to lack of students. Given the clear interest by the vast majority of providers we interviewed in learning Indigenous languages, it is unclear what barriers might exist in taking courses when available (e.g., financial, time, etc.).

### Planning to account for clinical needs from access to emergency situations

Providers also discussed strategies that consider that patients might not be able to attend all appointments and that resources needed during emergency situations will not always be available. For example, providers described efforts to schedule all appointments on the same day, given the limited socioeconomic resources of the population. One provider acknowledged that patients often face travel times over 6 hours to reach their appointment, and that many people from Chiapas neither own a car nor know how to drive, so they also have to find someone to take them to their appointment, pay them to drive, and also pay them for gasoline.

Another challenge in the region is poor access to blood banks, and any blood products available are often prioritized for obstetric hemorrhages. A workaround described by a provider involved prophylactically increasing a patient’s hemoglobin levels in prenatal visits via oral supplements and intramuscular iron injections in case of hemorrhage during surgery. The provider said, “At least three doses, and we are able to raise the hemoglobin between 1-1.5 points, which gives us a reserve during delivery in case there is hemorrhage.”

## Discussion

This study provides a comprehensive analysis of the factors that influence surgical care delivery in Chiapas, Mexico. Significant challenges exist with surgical referrals, within the local context, and in perioperative care. These challenges are compounded by inefficiencies in planning and management and by the complexities of a large public and private system. These barriers have led to innovations and modifications among those providing surgical care. Our results also have implications for strategies to strengthen surgical systems in low- and middle-income countries (LMICs).

While previous qualitative work has been done to evaluate healthcare delivery in Mexico, this is one of the first studies focusing specifically on surgical care. Several themes consistent with other studies were identified, including workforce and infrastructure shortages [16,17]. However, our results also identified themes not yet reported, including the maldistribution of surgical resources across hospitals and the bottleneck of surgical emergencies created at high level hospitals. Precise distribution of supplies, infrastructure, and workforce is critical in the field of surgery, where an operation can only be provided when all components of the surgical system are present. If any is missing, the remaining resources are not able to be utilized and become a source of inefficiency in the system.

**Fig 1:**
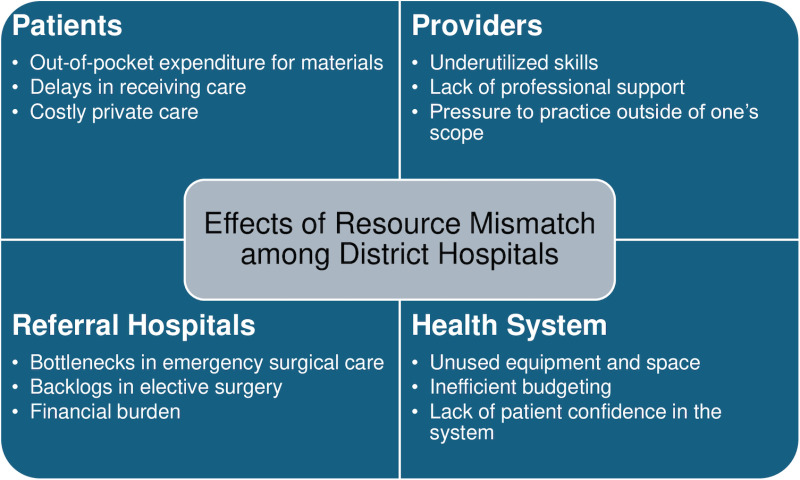
Effects of inefficient resource distribution among hospitals.

The mismatch in surgical resources impacts all aspects of healthcare (Fig 1). When the materials, diagnostic equipment, infrastructure, and healthcare specialists needed for a surgical procedure are not all present at the same hospital, patients must travel to a referral center. This increases out-of-pocket costs and leads to delays in care. Some patients may turn to private care which is more costly. And when providers are at a hospital without a complete surgical team, their skills are not able to be utilized. They also feel a lack of support and may be asked to practice outside of their normal scope. These inefficiencies have a downstream effect on the system, where referral hospitals receive many patients and are saturated with emergencies. This creates delays for elective surgeries and a lack of patient confidence in the system.

Causes of these barriers have deep roots in the system and are not only the result of inadequate system inputs. Historic conflicts in the region, language barriers, and cultural differences among communities create complex barriers that remain despite national policies providing insurance coverage or additional surgical staff and hospitals. Political pressures within the Ministry of Health also create incentives leading to policies that lack long-term, durable solutions benefitting the most marginalized populations [27,28]. Additionally, increasing the number of providers in rural areas cannot be accomplished by simply training more physicians. These barriers will require policymakers to rethink the way global surgery indicators are used in policy and targets are reached. Complex interactions between institutions and structures within the surgical ecosystem are also reflected in our results and will need further research. This includes tension between public and private systems, corruption, and inefficient resource distribution.

Qualitative studies investigating surgical care in LMICs from other regions of the world have yielded some consistencies with our results. Referral challenges have been reported in other regions of the world, including Ethiopia, Uganda, and South Asia [20,21,29,30]. Kelly and colleagues reported a lack of communication among referring providers in Ethiopia, while Albutt provided accounts of patients being referred due to lack of basic supplies such as sterile drapes or gowns [21,30]. In Uganda, receiving hospitals often did not have the capacity to provide the needed surgical care [20,21]. Examples of referral challenges in South Asia consisted of the burden patients face when transfer to a higher level of care is recommended, often precluding a successful referral [29]. While surgical workforce shortages in Mexico have been well documented in quantitative research, reasons for these gaps have been elucidated in this study and include low salaries and lack of support, as well as quality of life (both personal and professional) in resource constrained areas. Challenges recruiting surgical specialists to rural areas that stem from lack of support and low morale were also described by Albutt and colleagues. This included poor infrastructure and administrative oversight, high workload, and lack of continuing education [21]. In several regions of Africa and Andean Latin America, providers have described poor living conditions and feeling isolated in their practice settings in addition to underpayment and few opportunities for professional development [14,29]. Inefficiencies and lack of coordination in surgical systems have also been described in many other parts of the world. In Vanuatu, Young and colleagues provided examples of uneven distribution of surgical services and staff across the country, including the construction of new operating rooms and surgical space that was often unused due to adequate surgical providers and staff [31].

Given the challenges and salient themes described by providers, solutions that stem from the innovations and workarounds already in place and from examples in the literature could be expanded or standardized. To address the surgical workforce gap in rural areas, programs to improve the practice environment could be designed. These could be in the form of continuing education programs, mentorship from surgeons in urban areas, and support to attend conferences. Professional societies and nongovernmental organizations have engaged in these activities with positive results. For example, the Royal Australasian College of Surgeons Pacific Islands Program provides training and mentorship to local surgeons to build capacity that is aligned with national strategies and priorities [32]. Data on mentorship in LMICs to strengthen and retain workforce has also demonstrated its value. Shehnaz and colleagues conducted a mixed-methods evaluation of the impact of surgical mentorship in LMICs and found that it led to an increase in job satisfaction, mentee empowerment, a safer work environment, and a culture of continuous learning [33].

To overcome some of the challenges faced by indigenous populations, indigenous language training programs could be made more accessible and providers could engage with traditional healers and practitioners in rural communities. Providers in South Asia have described successful programs that involve meeting with traditional healers to provide education on surgical conditions, and in sub-Saharan Africa one government-sponsored program provided traditional midwives with formal training and sterile equipment [29]. In addition to interventions targeting providers, programs to assist patients navigating the health system have shown promise. In Mexico, Macias and colleagues described the value of accompaniment in the surgical referral process. Providers and patients have found that having a healthcare worker whose role is to guide and support a patient who needs surgery has mitigated the challenges within a complex health system and improves communication [34].

At the level of the health system, changes can be made to the way national surgical planning is approached. The NSOAP framework has been used in several countries and focuses heavily on system inputs (*e.g.*, equipment and infrastructure, surgical workforce, or funding mechanisms), however other frameworks may provide a more comprehensive understanding of the needs within a surgical ecosystem. For example, the World Health Organization Western Pacific Region employs an action framework for universal health coverage that emphasizes quality, efficiency, equity, accountability, and resilience. It aims to improve existing health system structures and processes to improve performance [35]. Another initiative that can inform surgical planning is the Lancet Commission on High Quality Health Systems, which provides tools to identify variation in healthcare quality while designing structural changes that improve patient experience and outcomes [36]. Our results point to key areas for improvement, especially as governments use these frameworks to prioritize efficiency and quality. By viewing surgical care as a system that can only function when all of the pieces are present, inefficiency and waste in systems can be found in areas where the surgical system is incomplete. This can lead to better allocation of resources so that hospital space, equipment, and workers do not go underutilized.

The results of this study have several implications for national surgical planning and creation of policies in global surgery. First, the surgical system domains are highly connected and interdependent, and this complexity should be considered when developing improvement strategies, which often focus on individual domains [14,37–39]. Second, vertical health system interventions cannot address the complex challenges of a surgical ecosystem. Comprehensive strategies with input from a variety of stakeholders are needed if the roots of these problems are to be identified and addressed. Third, NSOAPs and strategies should be tailored to the unique needs of the local population and system. Disparities within a country should be identified and NSOAPs should include provisions to address these at the local level. Finally, a patient-centered care approach is needed, particularly for indigenous populations to incorporate language barriers, cultural norms, and beliefs.

This study aimed to better understand the factors that influence surgical care delivery in Chiapas, Mexico, but limitations in our sample of hospitals must be acknowledged. Only hospitals from the private system and the public system for individuals without employment-based insurance were included. Themes identified in this study may not represent the challenges faced by providers in the employment-based healthcare systems. Despite this limitation, these results provide a robust analysis of the challenges and innovations that influence surgical care delivery in Chiapas, Mexico using qualitative analysis.

## Conclusion

This study describes the context in which surgical care is provided in public and private hospitals in Chiapas, across the domains of infrastructure, workforce, service delivery, financing, and information management. While we identified key barriers and challenges relevant to Chiapas, participants also described innovations and strategies already being employed locally. These innovations can inform solutions to improve surgical care for patients and highlight opportunities to build upon and adapt existing programs that have been successful elsewhere.

## Supporting information

S1 File Inclusivity in global research. (DOCX)
